# Chronic pancreatitis in *T7C140S* mice with misfolding cationic trypsinogen mutant

**DOI:** 10.1172/jci.insight.186516

**Published:** 2025-03-11

**Authors:** Máté Sándor, Balázs Csaba Németh, Alexandra Demcsák, Miklós Sahin-Tóth

**Affiliations:** Department of Surgery, UCLA, Los Angeles, California, USA.

**Keywords:** Gastroenterology, Genetics, Inflammation, Genetic diseases, Proteases, Protein misfolding

## To the Editor:

Hereditary chronic pancreatitis (CP) is caused by risk alleles that increase the intrapancreatic activation of the digestive protease cationic trypsinogen (PRSS1) to its active form trypsin (1). Increased trypsin activity in the pancreas drives CP onset and progression, as demonstrated by preclinical mouse models harboring mutant trypsinogens prone to increased autoactivation (2). Some patients with hereditary or sporadic CP, however, carry *PRSS1* mutations that diminish trypsinogen secretion and cause endoplasmic reticulum (ER) stress in cell culture experiments (3). This phenotype is reminiscent of the misfolding behavior of CP-associated carboxypeptidase A1 (*CPA1*) mutations (3, 4). Thus, we posit that a subset of *PRSS1* mutations may exert its effect via a mechanism that is unrelated to trypsin activity but involves trypsinogen misfolding and ER stress.

In the present study, we tested this hypothesis by generating the *T7C140S* mouse strain, which carries mutation p.C140S in the mouse cationic (T7) trypsinogen. This mutation is analogous to the CP-associated *PRSS1* mutation p.C139S, which was shown to exhibit the misfolding phenotype in cell culture (3). We also crossed the *T7C140S* mice with the *CPA1 N256K* strain, which carries the human p.N256K misfolding variant in the mouse *Cpa1* gene (4), to examine whether CP pathology can be amplified by coexpression of different misfolding enzymes. To accentuate the phenotype, all mice were homozygous. In our experiments, we documented the development of pancreas pathology and ER stress in *T7C140S*, *CPA1* *N256K*, and *T7C140S* × *CPA1* *N256K* mutant mice in comparison with C57BL/6N controls.

**Figure 1:**
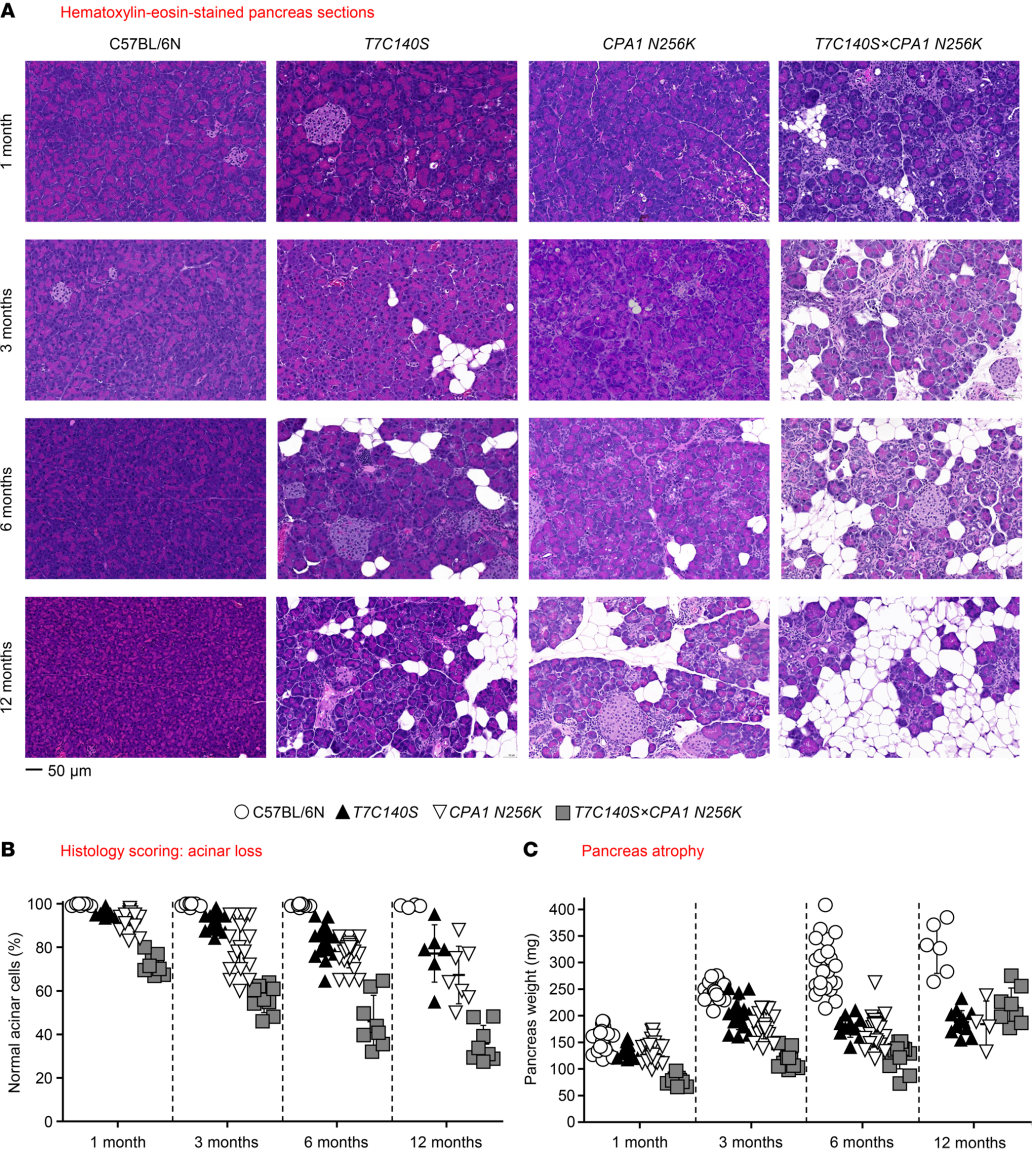
Pancreas histology and pancreas weight in C57BL/6N, *T7C140S*, *CPA1* *N256K*, and *T7C140S* × *CPA1* *N256K* mice. (**A**) Representative hematoxylin and eosin–stained pancreas sections. Scale bar: 50 μm. (**B**) Quantitation of acinar cell loss by histology scoring of pancreas sections. Individual values with the mean and standard deviation are shown. The number of sections evaluated, from left to right, were 10, 14, 25, 9, 10, 23, 18, 9, 10, 24, 20, 8, 8, 6, 7, and 8. Relative to C57BL/6N controls, the area of normal acini was significantly smaller in *T7C140S* mice (*P* = 0.0691 at 1 month, *P* = 0.035 at 3 months, *P* < 0.0001 at 6 months, *P* = 0.0229 at 12 months), *CPA1* *N256K* mice (*P* < 0.0001 at 1, 3, 6 months, *P* = 0.0006 at 12 months), and *T7C140S* × *CPA1* *N256K* mice (*P* < 0.0001 at all ages). (**C**) Pancreas weight in mg units. Individual values with the mean and standard deviation are shown. The number of pancreata measured, from left to right, were 23, 10, 25, 9, 15, 17, 16, 15, 25, 10, 25, 12, 10, 13, 4, and 8. Relative to C57BL/6N controls, the pancreas weight of *T7C140S*, *CPA1* *N256K*, and *T7C140S* × *CPA1* *N256K* mice was significantly lower at all ages (*P* < 0.0001), except for *T7C140S* (*P* = 0.0159) and *CPA1 N256K* (*P* = 0.0008) at 1 month. The difference of means was analyzed by 1-way ANOVA followed by Tukey’s post hoc test.

Pancreatic expression of cationic (T7) trypsinogen mRNA and protein in 1-month-old *T7C140S* mice was reduced by approximately 60% (see Methods) and approximately 30% (Supplemental Figure 1A; supplemental material available online with this article; https://doi.org/10.1172/jci.insight.186516DS1), respectively, relative to C57BL/6N animals. Total trypsinogen and chymotrypsinogen content were elevated in the pancreas of 1-month-old *T7C140S* and *CPA1* *N256K* mice versus C57BL/6N animals (Supplemental Figure 1A), whereas tissue amylase content was comparable (Supplemental Figure 1B). When the body weight of the 4 mouse lines was compared, no significant difference was seen at 1, 3, and 6 months of age, whereas a significant decrease was noted in the mutant strains relative to C57BL/6N mice at 12 months of age (Supplemental Figure 1C). Histological analysis of the pancreas by hematoxylin and eosin staining, immunohistochemistry (IHC), and Masson’s trichrome staining revealed progressive CP in mutant mice but not in C57BL/6N controls (Figure 1A and Supplemental Figure 1D). Disease severity and rate of progression were markedly higher in *T7C140S* × *CPA1* *N256K* mice compared with the parental strains (Figure 1A). Characteristic histological features of CP included acinar cell atrophy (Figure 1B), duct-like structures (SOX9 IHC, Supplemental Figure 1D), macrophage infiltration (F4/80 IHC, Supplemental Figure 1D), fibrosis (trichrome, Supplemental Figure 1D), adipose tissue replacement (Supplemental Figure 2A), and increased hydroxyproline content (Supplemental Figure 2B). Relative to C57BL/6N mice, the pancreas weight from the mutant strains was significantly reduced at all time points, with more severe atrophy seen with the compound genotype at 1, 3, and 6 months (Figure 1C). Analysis of pancreatic hydroxyproline content as a marker of collagen deposition revealed significantly increased fibrosis in *CPA1* *N256K* and *T7C140S* × *CPA1* *N256K* mice, but not in *T7C140S* mice when compared to C57BL/6N controls (Supplemental Figure 2B). Plasma amylase levels in *T7C140S* and *CPA1* *N256K* mice were significantly elevated at 1 and 3 months of age but were seemingly normal again at 6 and 12 months, likely due to the loss of acini at later ages (Supplemental Figure 2C). Intrapancreatic protease activation was assessed at 3 months of age. Significant elevation of trypsin activity was found only in the *T7C140S* × *CPA1* *N256K* strain, whereas chymotrypsin activity was increased significantly in *CPA1* *N256K* mice and to a nonsignificant degree in *T7C140S* and *T7C140S* × *CPA1* *N256K* mice (Supplemental Figure 2D). Finally, we measured pancreatic mRNA levels of the ER stress markers *Hspa5* (BiP) and *Ddit3* (CHOP) (Supplemental Figure 3). Compared with C57BL/6N mice, both transcripts were increased in the mutant strains at 3 and 6 months. At 1 month of age, *Hspa5* was elevated in *T7C140S* × *CPA1* *N256K* mice only, whereas increased *Ddit3* levels were detected in *CPA1* *N256K* and *T7C140S* × *CPA1* *N256K* animals. Since *T7C140S* mice showed no signs of ER stress at 1 month, we measured *XBP1* splicing in all 4 strains at this time point. Surprisingly, significant splicing was observed in *T7C140S* mice, while spliced *XBP1* levels in *CPA1* *N256K* and *T7C140S* × *CPA1* *N256K* animals were comparable to those seen in C57BL/6N controls (Supplemental Figure 3). These observations indicate that ER stress pathways become activated asynchronously in the mutant mouse strains, which likely reflects the variable degree of proteotoxicity in their pancreas.

In conclusion, our observations demonstrated that a misfolding trypsinogen mutant elicited progressive CP in mice, which phenocopied other mouse models expressing misfolding digestive enzymes (4–6). The data provide compelling evidence that misfolding *PRSS1* variants should be considered a separate etiological category that mechanistically resemble *CPA1* variants. Since antitrypsin therapy may not be effective in carriers of misfolding *PRSS1* mutations, new therapeutic approaches will have to target protein folding and ER stress pathways.

## Supplementary Material

Supplemental data

Unedited blot and gel images

Supporting data values

## Version 1 03/11/2025

In-Press Preview

## Version 2 04/22/2025

Electronic publication
