# Supplementary material for: Chronic pancreatitis in T7C140S mice with misfolding cationic trypsinogen mutant
Source: JCI Insight. 2025 Mar 11;10(8):e186516. doi: 10.1172/jci.insight.186516 (PMC12016918; doi:10.1172/jci.insight.186516)
Supplement: Supplemental data [file jciinsight-10-186516-s069.pdf]

SUPPLEMENTARY MATERIAL

Chronic pancreatitis in *T7C140S* mice with misfolding cationic trypsinogen mutant

Máté Sándor, Balázs Csaba Németh, Alexandra Demcsák, Miklós Sahin-Tóth

Department of Surgery, University of California Los Angeles, Los Angeles, California 90095

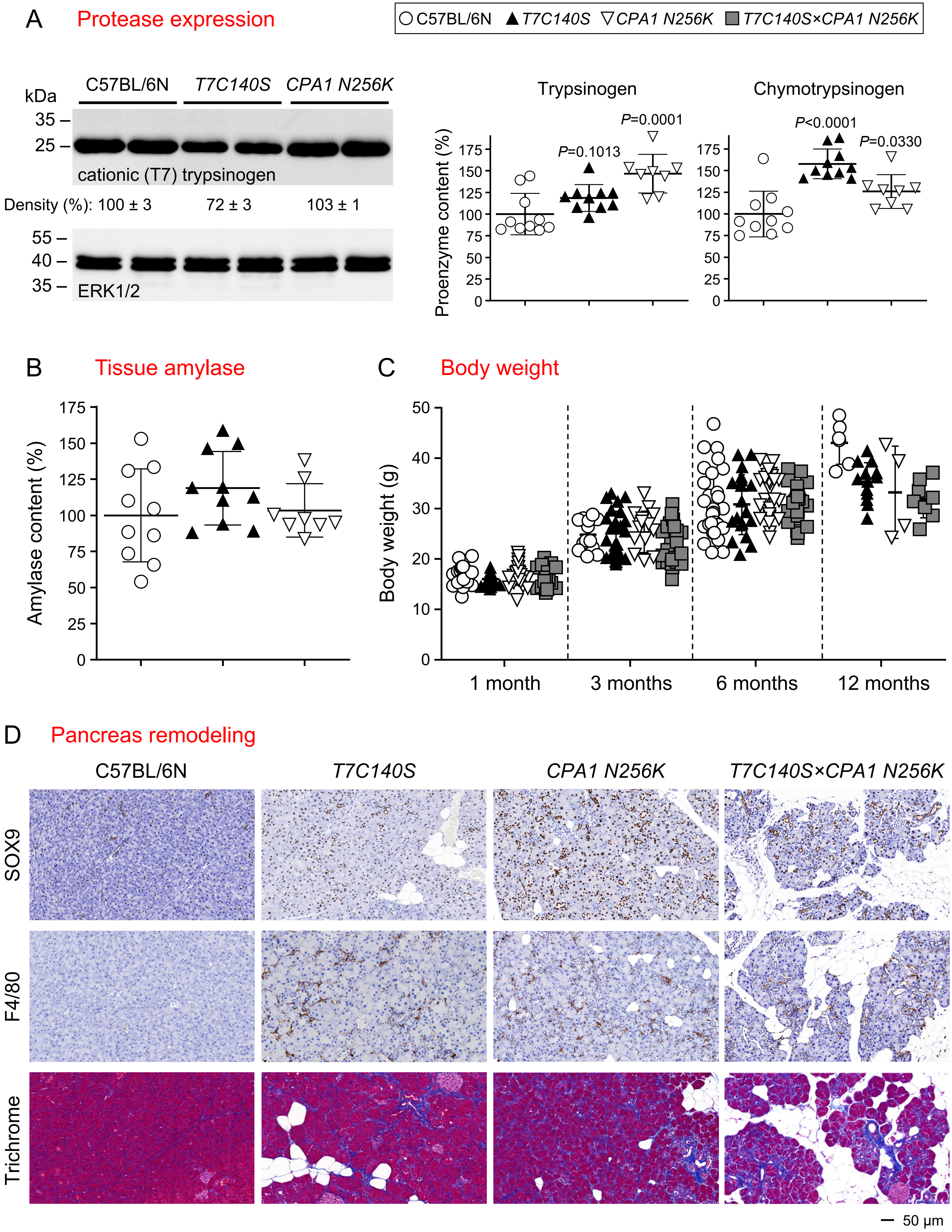
A Protease expression
C57BL/6N T7C140S CPA1 N256K T7C140S×CPA1 N256K
kDa
C57BL/6N T7C140S CPA1 N256K
35 –
25 –
cationic (T7) trypsinogen
Density (%): 100 ± 3 72 ± 3 103 ± 1
55 –
40 –
35 –
ERK1/2
Trypsinogen
Chymotrypsinogen
Proenzyme content (%)
P=0.1013
P=0.0001
P<0.0001
P=0.0330
B Tissue amylase
Amylase content (%)
C Body weight
Body weight (g)
1 month
3 months
6 months
12 months
D Pancreas remodeling
C57BL/6N
T7C140S
CPA1 N256K
T7C140S×CPA1 N256K
SOX9
F4/80
Trichrome
50 µm

Figure S1

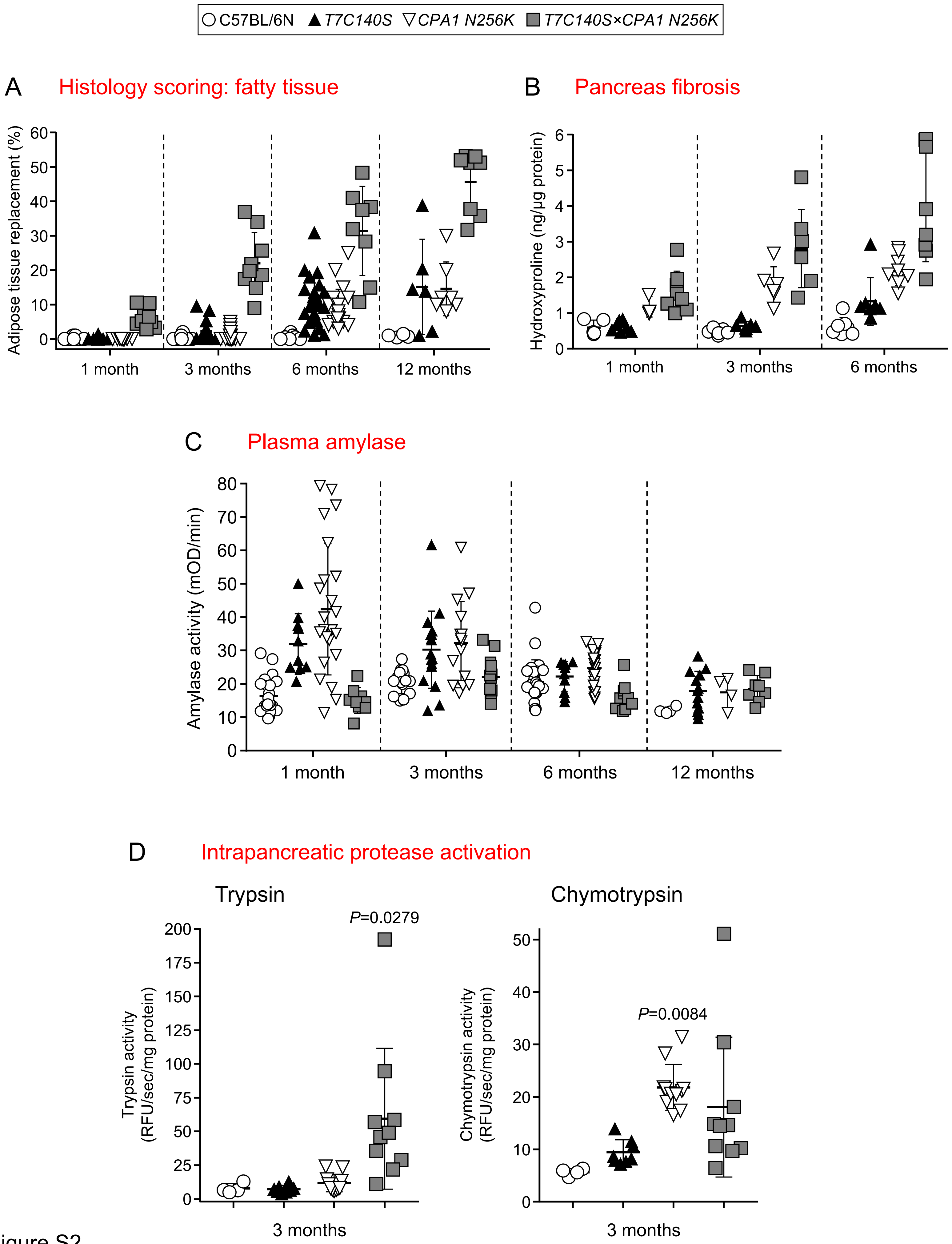

C57BL/6N
T7C140S
CPA1 N256K
T7C140S×CPA1 N256K
A
Histology scoring: fatty tissue
Adipose tissue replacement (%)
1 month
3 months
6 months
12 months
B
Pancreas fibrosis
Hydroxyproline (ng/µg protein)
1 month
3 months
6 months
C
Plasma amylase
Amylase activity (mOD/min)
1 month
3 months
6 months
12 months
D
Intrapancreatic protease activation
Trypsin
P=0.0279
Trypsin activity (RFU/sec/mg protein)
3 months
Chymotrypsin
P=0.0084
Chymotrypsin activity (RFU/sec/mg protein)
3 months

Figure S2

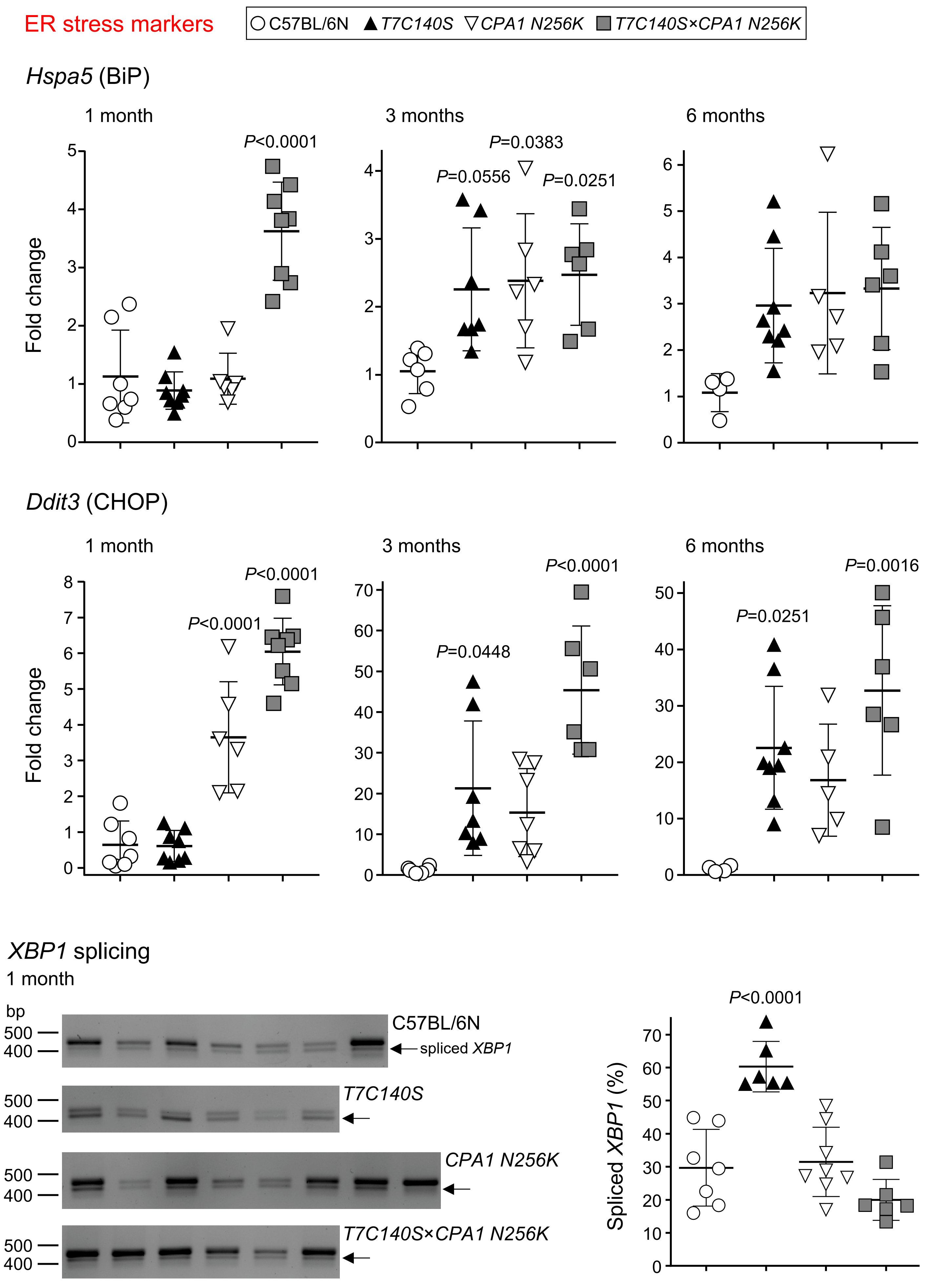
ER stress markers
C57BL/6N
T7C140S
CPA1 N256K
T7C140S×CPA1 N256K
Hspa5 (BiP)
1 month
3 months
6 months
Fold change
P<0.0001
P=0.0383
P=0.0556
P=0.0251
Ddit3 (CHOP)
P<0.0001
P<0.0001
P<0.0001
P=0.0448
P=0.0016
P=0.0251
XBP1 splicing
bp
500
400
spliced XBP1
Spliced XBP1 (%)
P<0.0001

Figure S3

LEGENDS TO SUPPLEMENTARY FIGURES

Supplementary Figure S1. Pancreatic protease zymogen expression, tissue amylase content, body weight, and pancreas remodeling in C57BL/6N, *T7C140S*, *CPA1 N256K*, and *T7C140S×CPA1 N256K* mice. Where applicable, individual values with the mean and standard deviation are shown. The difference of means was analyzed by one-way ANOVA followed by Tukey's post-hoc test. Pairwise comparison was performed against C57BL/6N. **A**, Western blot analysis of mouse cationic (T7) trypsinogen and assay of total trypsinogen and chymotrypsinogen content in pancreas homogenates from 1-month-old C57BL/6N, *T7C140S,* and *CPA1 N256K* mice. Proenzyme levels were normalized to protein content and expressed in percentage of the mean C57BL/6N data. **B**, Tissue amylase content in 1-month-old C57BL/6N, *T7C140S,* and *CPA1 N256K* mice. Amylase levels were normalized to protein content and expressed in percentage of the mean C57BL/6N data. **C**, Body weight in g units as a function of age. The number of mice measured, from left to right, were 29, 19, 25, 18, 15, 28, 16, 25, 30, 21, 25, 23, 10, 13, 4, 8. Relative to C57BL/6N controls, the body weight of *T7C140S, CPA1 N256K,* and *T7C140S×CPA1 N256K* mice was significantly lower at 12 months of age (P=0.014, P=0.0173, and P<0.0009, respectively). **D**, Immunohistochemistry (IHC) and Masson's trichrome staining of pancreas sections from 6-month-old mice. IHC for SOX9 labels ducts and duct-like cells (e.g. acinar-to-ductal metaplasia), IHC positivity for F4/80 represents macrophage infiltration, and trichrome staining highlights fibrosis in blue color.

Supplementary Figure S2. Pancreas remodeling, plasma amylase, and intrapancreatic protease activity in C57BL/6N, *T7C140S*, *CPA1 N256K*, and *T7C140S×CPA1 N256K* mice. Individual values with the mean and standard deviation are shown. The difference of means was analyzed by one-way ANOVA followed by Tukey's post-hoc test. Pairwise comparison was performed against C57BL/6N. **A,** Adipose replacement of acinar tissue as a function of age. Hematoxylin-eosin-stained pancreas sections were evaluated for fatty infiltration, which was quantitated as percent of the tissue area examined. The number of sections assessed, from left to right, were 10, 14, 25, 9, 10, 23, 18, 9, 10, 24, 20, 8, 8, 6, 7, 8. Relative to C57BL/6N controls, fatty replacement in *T7C140S* and *CPA1 N256K* mice was significantly higher at 6 months of age (P=0.002 and P=0.028, respectively), and in the *T7C140S×CPA1 N256K* mutant strain at all ages (P<0.0001). **B**, Hydroxyproline content of pancreas homogenates as a function of age. Increased hydroxyproline levels signify collagen deposition (fibrosis). The number of samples measured, from left to right,

were 5, 8, 8, 7, 7, 7, 7, 8, and 8. Relative to C57BL/6N controls, the pancreatic hydroxyproline content was significantly higher in *CPA1 N256K* mice at 3 months (*P*=0.0042) and 6 months of age (*P*=0.0084) and in *T7C140S×CPA1 N256K* mice at all ages (*P*=0.0007 at 1 month, *P*<0.0001 at 3 and 6 months). **C**, Plasma amylase activity. The number of mice studied, from left to right, were 23, 10, 25, 9, 15, 17, 15, 15, 25, 10, 21, 12, 8, 13, 4, 8. Relative to C57BL/6N controls, plasma amylase levels in *T7C140S* and *CPA1 N256K* mice were significantly higher at 1 month (*P*=0.0113 and *P*<0.0001) and 3 months of age (*P*=0.0238 and *P*=0.0054). **D**, Intrapancreatic trypsin and chymotrypsin activity in 3-month-old mice. The number of pancreas homogenates analyzed, from left to right, were 4, 8, 11, 10.

Supplementary Figure S3. Endoplasmic reticulum (ER) stress in the pancreas of C57BL/6N, *T7C140S*, *CPA1 N256K*, and *T7C140S×CPA1 N256K* mice. Individual values with the mean and standard deviation are shown. The difference of means was analyzed by one-way ANOVA followed by Tukey's post-hoc test. Pairwise comparison was performed against C57BL/6N. Messenger RNA expression of ER stress markers *Hspa5* (BiP) and *Ddit3* (CHOP) was measured by reverse transcription quantitative PCR at 1, 3, and 6 months of age. Results were expressed as fold change relative to the mean of the C57BL/6N data. Splicing of *XBP1* in the pancreas of 1-month-old mice was analyzed by reverse transcription PCR, agarose gel electrophoresis (left panel), and densitometry (right panel). Results were expressed in percentage of the sum of the unspliced (upper) and spliced (lower) bands. The arrows indicate the spliced form.

METHODS

Accession numbers and nomenclature. NC_000072.7, *Mus Musculus* strain C57BL/6J chromosome 6 genomic sequence; NM_023333.4, *Mus musculus* serine protease 3B (*Prss3b*) mRNA sequence, formerly known as RIKEN cDNA 2210010C04 (2210010C04Rik) mRNA, encoding the mouse cationic (T7) trypsinogen. Nucleotides in the coding sequence of trypsinogen were numbered starting from the first nucleotide of the ATG translation initiator codon. Amino-acid residues in trypsinogen were numbered starting from the initiator methionine. Note that amino-acid numbering of mouse cationic (T7) trypsinogen is shifted by 1 relative to human trypsinogens due to an extra Asp residue in the activation peptide. Thus, mutation p.C139S in human cationic trypsinogen corresponds to p.C140S in mouse cationic (T7) trypsinogen.

Mouse strains. Generation of the novel *T7C140S* strain was carried out on the C57BL/6N genetic background (Cyagen, Santa Clara, CA) using the general strategy and protocols described for various other mouse strains carrying mutations in mouse cationic (T7) trypsinogen (1-4). The final *T7C140S* knock-in allele contained the c.418T>A (p.C140S) mutation in exon 3 of the mouse cationic (T7) trypsinogen gene and a 113 nt residual "scar" sequence in intron 2. Owing to these genomic changes, pancreatic mRNA levels of mouse cationic (T7) trypsinogen in 1-month-old *T7C140S* mice were lower (41 ± 14%, mean ± standard deviation, n=6) relative to C57BL/6N mice, as judged by reverse transcription quantitative PCR. Generation and properties of the *CPA1 N256K* mouse strain (Cyagen, Santa Clara, CA) carrying the human p.N256K mutation in the mouse *Cpa1* gene were reported previously (5). The *T7C140S×CPA1 N256K* strain was created by crossing the respective parent strains. Even though both genes are located on chromosome 6, we obtained pups with the compound allele. All mutant mice were maintained and used in the homozygous state. C57BL/6N mice obtained from Charles River Laboratories (Wilmington, MA) or produced in our breeding facility from the same stock were used as experimental controls. The number of experimental animals is shown on the figures or described in the figure legends. In keeping with the principles of the 3Rs (replace, reduce, refine), most of the C57BL/6N and *CPA1 N256K* mice were used for multiple projects at the same time and some data were already reported (5).

Sex as a biological variable. Both male (55%) and female (45%) animals were studied. Subgroup analysis of the experimental data revealed no significant sex-specific differences in the severity of

chronic pancreatitis in *T7C140S*, *CPA1 N256K*, and *T7C140S×CPA1 N256K* mice (reviewed but not shown).

Genotyping. To genotype the *T7C140S* allele, we used PCR primers flanking the "scar" sequence in intron 2. Forward primer, 5'- TCC ACT GGG CAT CTG CAT TCA TAC -3′, reverse primer, 5′- GCA GAG CAA TGT TAT AGG ACA GAG -3′. The PCR products were analyzed by agarose gel electrophoresis. The amplicon size from the wild-type allele was 367 bp, whereas the mutant allele yielded a 480 bp product due to the presence of the residual sequence in intron 2. For additional verification, we also used primers flanking the mutation to amplify a 425 bp sequence, which was then subjected to Sanger sequencing. The primers were: forward primer, 5'- CCT GGG AGA ACA CAA CAT TGA TGC -3', reverse primer, 5'- TGT TGT CTC TAT TGT ATG ATG TGG GCA -3'. Genotyping of the *CPA1 N256K* allele was reported previously (6).

Histology. Pancreas tissue was fixed in 10% neutral buffered formalin, paraffin-embedded, sectioned and stained with hematoxylin-eosin, Masson's trichrome, or with immunohistochemistry for the ductal marker SOX9 and the macrophage marker F4/80 (Department of Pathology and Laboratory Medicine, Cedars-Sinai Medical Center, Los Angeles, CA, and UCLA Translational Pathology Core Laboratory). Hematoxylin-eosin-stained pancreas sections were scored by visual inspection for acinar atrophy and adipose infiltration, and the results were expressed in percentage of the total tissue area examined.

Hydroxyproline assay. Collagen deposition (fibrosis) was characterized by determining the hydroxyproline content of the pancreas, as described previously (6). Results were expressed in units of ng hydroxyproline per µg protein.

Plasma amylase assay. Blood was obtained by cardiac puncture in heparinized syringes. Cellular elements were separated from the plasma by centrifugation (2,000 g, 15 min, 4°C). Enzyme activity of amylase in blood plasma was determined using the 2-chloro-p-nitrophenyl-α-D-maltotrioside substrate (catalog number A7564-60; Pointe Scientific, Canton, MI) in a kinetic assay. An aliquot (1 µL) of plasma was diluted with 9 µL normal saline, and 190 µL substrate was added to initiate the reaction. Substrate cleavage was monitored at 405 nm for 2 min in a microplate reader, and the rate was expressed in mOD/min unit.

Tissue amylase assay. Pancreatic amylase content was determined from homogenates. Pancreas tissue (30-40 mg) was homogenized in 300-400 µL ice-cold 20 mM Na-HEPES (pH 7.4) buffer

and centrifuged at 850*g* for 10 minutes (4ºC) to remove heavy particulate matter. The cleared homogenate was diluted 100× with 20 mM Na-HEPES (pH 7.4) buffer and amylase activity was measured using 1 μL of diluted homogenate, as described for plasma amylase. The results were normalized to the protein content and expressed in percentage of the mean of the C57BL/6N data.

Measurement of pancreatic protease expression. The trypsinogen and chymotrypsinogen content of the pancreas was estimated from the trypsin and chymotrypsin activity measured after maximal activation, as reported previously (7). The rate of substrate cleavage was normalized to the total protein content and expressed in percentage of the mean value from C57BL/6N mice.

Intrapancreatic protease activity. Intrapancreatic trypsin and chymotrypsin activity was measured from freshly prepared pancreas homogenates according to our published protocol (8). Protease activity was expressed in RFU/sec/mg protein units, which represents the rate of substrate cleavage in relative fluorescent units per second, normalized to the total protein content.

Reverse transcription quantitative PCR. Total RNA was extracted and reverse-transcribed as reported previously (1). Expression levels of *Hspa5* (BiP) and *Ddit3* (CHOP) mRNA were then determined using TaqMan Gene Expression Assays (catalog numbers Mm00517691_m1 and Mm01135937_g1) and TaqMan Universal PCR Master Mix (catalog number 4304437, Thermo Fisher Scientific). The mouse *Rpl13a* (ribosomal protein L13a) was used as reference gene (catalog number Mm01612987_g1). Expression was quantitated using the comparative cycle threshold method (ΔΔCT method), as described (2).

Measurement of *XBP1* mRNA splicing. The spliced (421 bp) and unspliced (447 bp) forms of the mouse X-box binding protein 1 (*XBP1)* were co-amplified from pancreatic RNA using reverse transcription PCR. The following primers were used: *XBP1* sense primer, 5′- CCT TGT GGT TGA GAA CCA GG -3′; *XBP1* antisense primer, 5′- AGG CTT GGT GTA TAC ATG G -3′. The amplicons were resolved on 2.5% agarose gels and stained with GreenGlo Safe DNA Dye (catalog number C788T73, Thomas Scientific, Swedesboro, NJ). Densitometry of the spliced and unspliced band intensities was performed with the ImageJ software (version 1.52a). The amount of spliced *XBP1* was expressed in percentage of the sum of the spliced and unspliced bands.

Western blotting. Levels of mouse cationic (T7) trypsinogen protein in pancreas homogenates were assessed by western blotting using a rabbit polyclonal antibody at 1:10,000 dilution, as reported recently (9). The generation and validation of the antibody was described previously (1).

As loading control, ERK1/2 was measured using a rabbit monoclonal antibody at 1:1,000 dilution (catalog number 4695, Cell Signaling Technology, Danvers, MA). As secondary antibody, horseradish peroxidase-conjugated goat anti-rabbit IgG was used at 1:10,000 dilution (catalog number HAF008, R&D Systems, Minneapolis, MN).

Statistics. Experimental results were graphed as individual data points with the mean and standard deviation values indicated. The difference of means was analyzed by one-way ANOVA followed by Tukey's post-hoc test. Pairwise comparison was performed against C57BL/6N. $P<0.05$ was considered statistically significant.

Study approval. Animal experiments were performed at the University of California, Los Angeles (UCLA) with the approval and oversight of the Animal Research Committee, including protocol review and post-approval monitoring. The animal care program at UCLA is managed in full compliance with the US Animal Welfare Act, the United States Department of Agriculture Animal Welfare Regulations, the US Public Health Service Policy on Humane Care and Use of Laboratory Animals and the National Research Council's Guide for the Care and Use of Laboratory Animals. UCLA has an approved Animal Welfare Assurance statement on file with the US Public Health Service, National Institutes of Health, Office of Laboratory Animal Welfare, and it is accredited by the Association for Assessment and Accreditation of Laboratory Animal Care International (AAALAC).

Data availability. All data are available from the corresponding author upon request. Data values used for the figures are listed in the "Supporting data values" file.

REFERENCES

1. Geisz A, Sahin-Tóth M. A preclinical model of chronic pancreatitis driven by trypsinogen autoactivation. *Nat Commun*. 2018;9(1):5033.

2. Jancsó Z, Sahin-Tóth M. Mutation that promotes activation of trypsinogen increases severity of secretagogue-induced pancreatitis in mice. *Gastroenterology*. 2020;158(4):1083-1094.

3. Demcsák A, Sahin-Tóth M. Rate of autoactivation determines pancreatitis phenotype in trypsinogen mutant mice. *Gastroenterology*. 2022;163(3):761-763.

4. Jancsó Z, et al. Mouse model of *PRSS1* p.R122H-related hereditary pancreatitis highlights context-dependent effect of autolysis-site mutation. *Pancreatology*. 2023;23(2):131-142.

5. Németh BC, et al. Misfolding-induced chronic pancreatitis in *CPA1 N256K* mutant mice is unaffected by global deletion of *Ddit3/Chop*. *Sci Rep*. 2022;12(1):6357.

6. Hegyi E, Sahin-Tóth M. Human *CPA1* mutation causes digestive enzyme misfolding and chronic pancreatitis in mice. *Gut*. 2019;68(2):301-312.

7. Demcsák A, Shariatzadeh S, Sahin-Tóth M. Secretagogue-induced pancreatitis in mice devoid of chymotrypsin. *Am J Physiol Gastrointest Liver Physiol*. 2024;327(3):G333-G344.

8. Mosztbacher D, Demcsák A, Sahin-Tóth M. Measuring digestive protease activation in the mouse pancreas. *Pancreatology*. 2020;20(2):288-292.

9. Demcsák A, Sahin-Tóth M. Heterozygous *Spink1* deficiency promotes trypsin-dependent chronic pancreatitis in mice. *Cell Mol Gastroenterol Hepatol*. 2024;18(3):101361.

ACKNOWLEDGEMENTS

This work was supported by the National Institutes of Health (NIH) grants R01 DK117809 and R01 DK082412 to MST, the National Pancreas Foundation award 20233127, the Hirshberg Foundation for Pancreatic Cancer Research award 20230501, and the Department of Defense grant HT9425-24-1-0164 (PR230469) to AD, and a scholarship award from the Rosztoczy Foundation to BCN. The authors are grateful to Franziska Thiel and Zsanett Jancsó for their help with colony management and preliminary analyses.
