# Supplementary material for: Chronic pancreatitis in T7C140S mice with misfolding cationic trypsinogen mutant
Source: JCI Insight. 2025 Mar 11;10(8):e186516. doi: 10.1172/jci.insight.186516 (PMC12016918; doi:10.1172/jci.insight.186516)

Figure S1A, uncropped blot

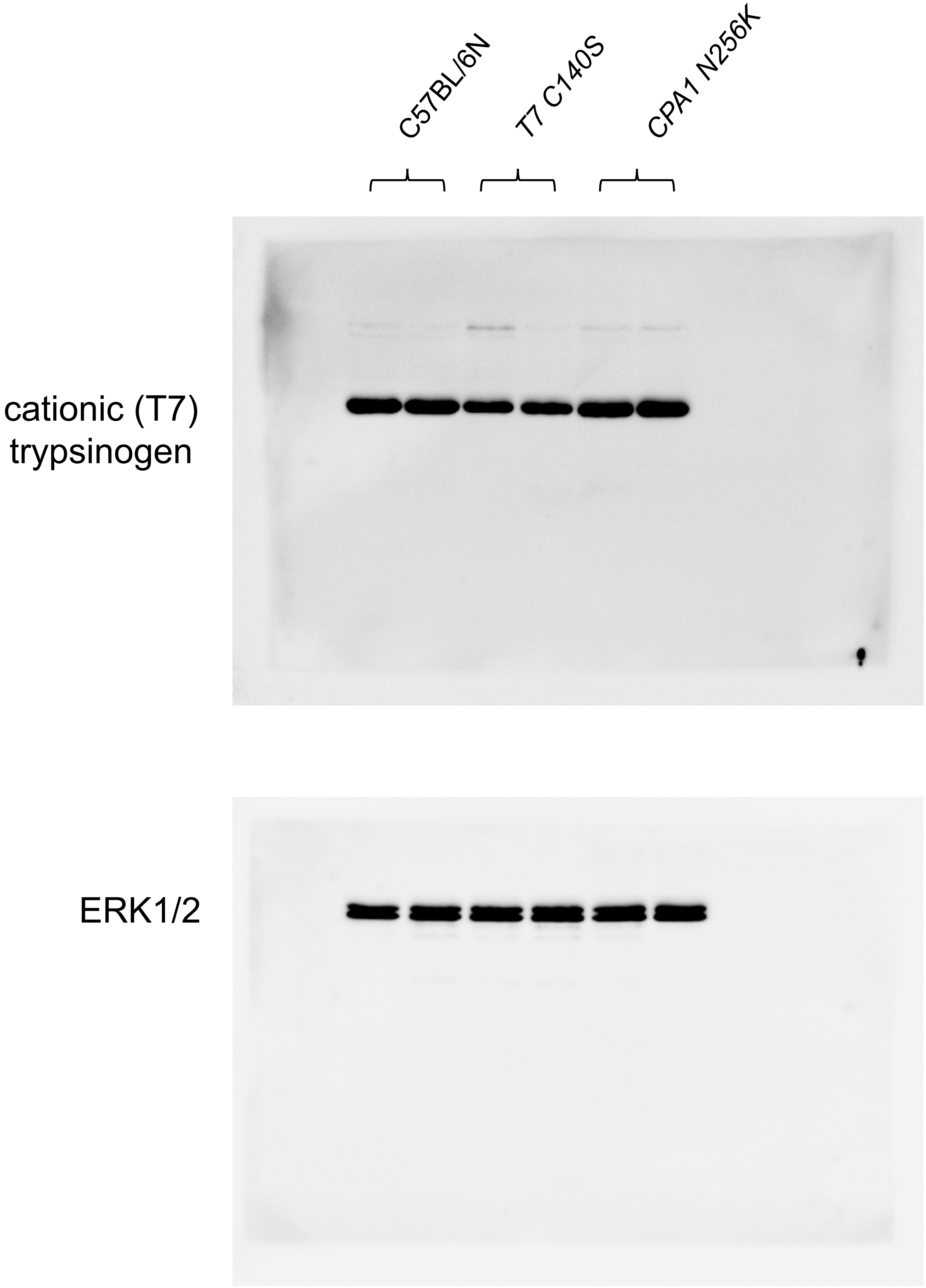

Figure S3, uncropped agarose gel pictures

100 bp DNA Ladder (Thermo Fisher Scientific, catalog number 15628050)

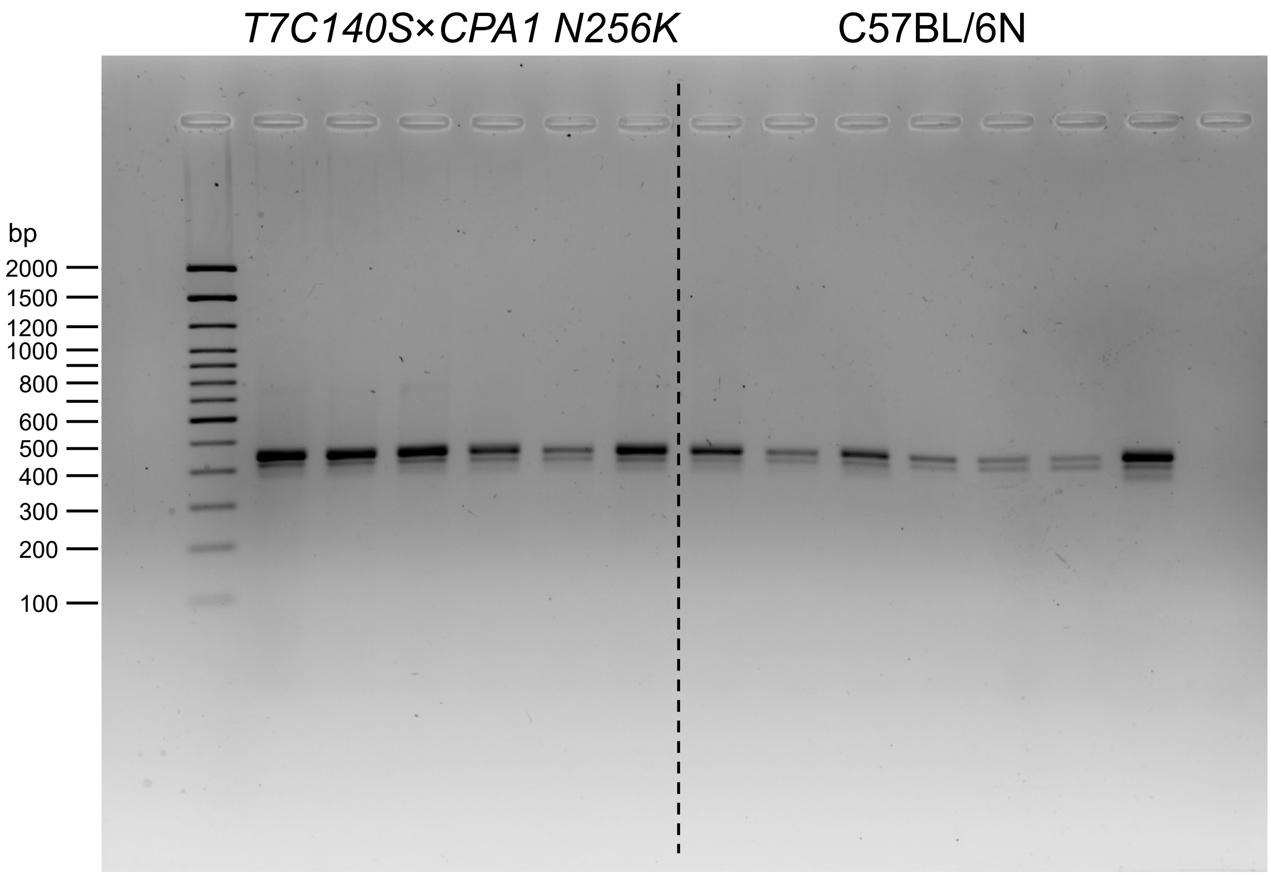

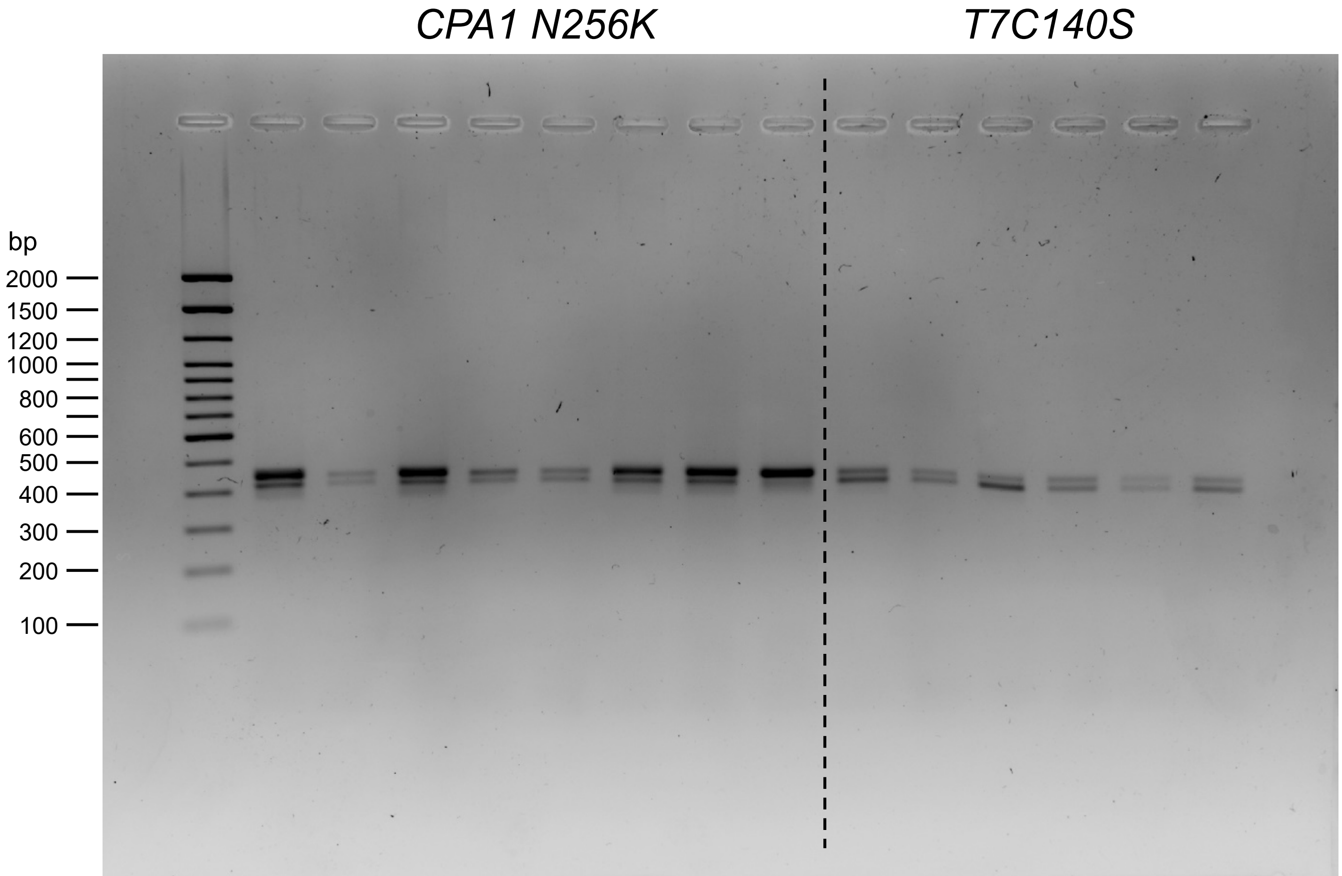

Supplement: Unedited blot and gel images [file jciinsight-10-186516-s070.pdf]
